# Intravoxel incoherent motion diffusion-weighted MRI for predicting the efficacy of high-intensity focused ultrasound ablation for uterine fibroids

**DOI:** 10.3389/fonc.2023.1178649

**Published:** 2023-06-22

**Authors:** Yu Jiang, Shize Qin, Yanlin Wang, Yang Liu, Nian Liu, Lingling Tang, Jie Fang, Qing Jia, Xiaohua Huang

**Affiliations:** ^1^ Department of Radiology, Affiliated Hospital of North Sichuan Medical College, Nanchong, China; ^2^ Department of Clinical Medicine, North Sichuan Medical College, Nanchong, China; ^3^ Department of Urology, Affiliated Hospital of North Sichuan Medical College, Nanchong, China

**Keywords:** uterine fibroid, high-intensive focused ultrasound, magnetic resonance imaging, intravoxel incoherent motion, diffusion-weighted imaging

## Abstract

**Purpose:**

To evaluate the significance of magnetic resonance (MR) intravoxel incoherent motion diffusion-weighted imaging (IVIM-DWI) quantitative parameters in predicting early efficacy of high-intensity focused ultrasound (HIFU) ablation of uterine fibroids before treatment.

**Method:**

64 patients with 89 uterine fibroids undergoing HIFU ablation (51 sufficient ablations and 38 insufficient ablations) were enrolled in the study and completed MR imaging and IVIM-DWI before treatment. The IVIM-DWI parameters, including D (diffusion coefficient), D^*^ (pseudo-diffusion coefficient), f (perfusion fraction) and relative blood flow (rBF) were calculated. The logistic regression (LR) model was constructed to analyze the predictors of efficacy. The receiver operating characteristic (ROC) curve was drawn to assess the model’s performance. A nomograph was constructed to visualize the model.

**Results:**

The D value of the sufficient ablation group (931.0(851.5-987.4) × 10^−6^ mm^2^/s) was significantly lower than that of the insufficient ablation group (1052.7(1019.6-1158.7) × 10^−6^ mm^2^/s) (*p*<0.001). However, differences in D^*^, f, and rBF values between the groups were not significant (*p*>0.05). The LR model was constructed with D value, fibroid position, ventral skin distance, T2WI signal intensity, and contrast enhanced degree. The area under the ROC curve, specificity, and sensitivity of the model were 0.858 (95% confidence interval: 0.781, 0.935), 0.686, and 0.947. The nomogram and calibration curves confirmed that the model had excellent performance.

**Conclusion:**

The IVIM-DWI quantitative parameters can be used to predict early effects of HIFU ablation on uterine fibroids. A high D value before treatment may indicate that the treatment will be less effective in the early stages.

## 1 Introduction

Uterine fibroids, defined as benign tumors formed due to hyperplasia of uterine smooth muscle tissues, are the most common pelvic benign tumors in women of reproductive age (1). Most women have no symptoms, in others, the primary symptoms include menstrual changes, pelvic pressure, pain, and negative fertility, which seriously affects the quality of life of patients (2, 3). Medical interventions are particularly important for symptomatic uterine fibroids. Clinically, the primary management strategies for uterine fibroids include surgical treatment, minimally invasive or non-invasive treatment, and drug treatment (4). Surgical treatment approaches, such as myomectomy and hysterectomy, are the most common treatment methods for women with uterine fibroids (5). However, they are associated with several limitations, including contraindications, long hospital stay time, postoperative complications, and the risk of fertility loss (6). Drug treatment is also limited by their inability to completely relieve symptoms or their associated side effects use (7). Advances in science and technology have resulted in the development of minimally invasive or non-invasive therapies such as uterine artery embolization (UAE) and high-intensity focused ultrasound (HIFU) for the symptomatic treatment of uterine fibroids (2). Clinically, UAE has the risk of post embolism syndrome development, and part of its efficacy may be attributed to impaired ovarian reserves (8, 9).

Recently, HIFU was proven to be effective as a non-invasive ablation modality for soft tissues. Moreover, HIFU ablation for uterine fibroids has the advantages of safety, accuracy, fertility retention, and quick recovery (10–13). It is an attractive treatment modality for many patients with uterine fibroids, including those who require preservation of the uterus and future fertility. It is also suitable for those who cannot tolerate or are unwilling to receive surgical treatments (14–16). Since the clinical results of HIFU ablation differ among patients with uterine fibroids, not all fibroids are suitable for HIFU treatment (17). Preoperative individualized efficacy prediction is important for accurate selection of patients, ensuring the success rate of HIFU treatment and saving medical costs (18, 19).

Magnetic resonance imaging (MRI) is usually performed to assess a patient’s suitability for a specific treatment (17, 20). The HIFU ablation technique has poor effects on uterine fibroids that are resistant to HIFU heating. Resistance is attributed to the high abundance of smooth muscle cells and less collagen fibers in fibroid tissue and to the high level of tissue perfusion of fibroids (17, 21). The two resistance factors show high signal intensity in T2-weighted imaging (T2WI) MRI sequence and obvious enhancement in contrast enhanced sequence (22, 23). However, these evaluation methods are often unreliable because they can be affected by the scanning parameters used and the experience of the observer. This can lead to inaccurate and unstable predictions of treatment outcomes (24). Evaluation of efficacy based on conventional T2WI may be limited by the limitations of T2WI in assessing blood vessels and blood perfusion of uterine fibroids. Moreover, there is a certain overlap in evaluating uterine fibroids of different pathological types (24, 25). Contrast enhanced MRI can be used to study the microvascular structure and functions, but it may have safety risks that are related to the contrast agent (17, 26).

Intravoxel incoherent motion diffusion-weighted imaging (IVIM-DWI) is a concept that was proposed by Le Bihan et al. (27). According to the IVIM-DWI model, diffusion and perfusion can be quantified using multi b-value DWI acquisition, and low b-value provides a higher sensitivity to perfusion (28). It can be used for non-invasive quantitative evaluation of microcirculation in capillaries and molecular diffusion, and is widely used in early detection, diagnosis, staging, monitoring progress and efficacy evaluation of diseases (28, 29). Moreover, IVIM-DWI mainly quantifies three parameters: the D value reflects real diffusion information, D^*^ value reflects perfusion information, and f value reflects the proportion of perfusion effects in total diffusion effect. The DWI can effectively assess ablation efficacies for most patients with uterine fibroids (30). Besides, the apparent diffusion coefficient (ADC) and D value of fibroids with high signals on T2WI before HIFU ablation are significantly higher than those of fibroids with low signals (31). Based on outcomes of these studies, we postulated that quantitative parameters derived from IVIM-DWI can predict early efficacy of HIFU ablation of uterine fibroids, which has been proven to be associated with the density of fibroid cells and blood supply. The purpose of this study was to explore the value of IVIM-DWI quantitative parameters in predicting the early efficacy of HIFU ablation of uterine fibroids before treatment.

## 2 Materials and methods

### 2.1 Study participants

This retrospective study was conducted in accordance with the Helsinki Declaration. The institutional review board approved this study (Approval No. 2022ER360-1). The requirement for informed consent was waived. A total of 113 patients with uterine fibroids undergoing HIFU ablation in our hospital were enrolled from October 2021 to October 2022.

The inclusion criteria were: i. Premenopausal or perimenopausal women aged over 18 years old that had been diagnosed with uterine fibroids; ii. Patients for whom HIFU ablation had been performed for the first time, and the number of fibroids was no more than 5, with a diameter of ≥ 3 cm and ≤ 10 cm (32); iii. Pelvic MRI scanning (including multi b value DWI) was performed within three days before and after HIFU ablation. The exclusion criteria were: i. Incomplete MR sequences or images (N=20); ii. Poor image quality (N=11); iii. Large areas of necrotic tissues in uterine fibroids (N=9); iv. The presence of skin ulcerations or infections in treatment-related areas, or abdominal scars (33) (N=2); and v. The presence of other solid lesions or non-benign lesions within the uterus and adnexa (N=7). Finally, 89 uterine fibroids (n=89) in 64 women (N=64; mean age, 43.6 ± 6.1 years) treated with HIFU ablation were analyzed.

A higher non-perfusion volume ratio (NPVR) after uterine fibroid ablation treatment is associated with symptomatic relief and reduced fibroid volume (34). The NPVR of uterine fibroids is defined as the volume of non-perfusion (NPV) tissue after treatment divided by the volume (V) of fibroids before treatment. The V and NPV were obtained using the ellipsoid volume calculation formula (volume = 0.5233 × L × W × D, L: length; W: width; and D: depth) on contrast enhanced T1-weighted imaging (T1WI) before and after ablation of uterine fibroids (35). Given that fibroids with a NPVR of more than 70% are significantly reduced in size within one year after treatment, 89 uterine fibroids were divided into a sufficient ablation group (NPVR≥70%, n=51) and an insufficient ablation group (NPVR <70%, n=38) using 70% NPVR as the reference (12, 36, 37).

### 2.2 The MRI assay

The MRI assay of the female pelvic regions of all study participants to assess uterine fibroids was performed using the 3.0T system (China, Unite Imaging Healthcare, uMR790) with a combination of 16-channel body and spine matrix coils. Each participant was placed in the supine position with head enter first. Then, a sandbag was placed on the abdomen to reduce respiratory artifacts.

The MRI sequences consisted of transverse T1WI, transverse T2WI, and transverse IVIM-DWI. The transverse IVIM-DWI sequence was on the basis of free-breathing spin echo planar imaging with the following parameters: the gradient was applied in the three orthogonal diffusion gradient directions of x, y, and z axes with 11 b values (0, 10, 25, 50, 80, 100, 150, 200, 500, 800, and 1000 s/mm^2^); the corresponding number of excitations of b values were 1, 1, 1, 1, 1, 1, 1, 2, 3, 4, and 5; repetition time/echo time, 2500/56 ms; scanning slice thickness, 4.0 mm; reconstructed slice thickness, 1.7 mm; no gap; field of view, 235 mm× 377 mm; scanning matrix, 56 × 112; reconstructed matrix, 112×224; flip angle, 90 degrees; generalized autocalibrating partially parallel acquisition; acceleration factor, 2; chemical shift selective fat suppression technique.

### 2.3 Baseline data collection

Baseline data of uterine fibroids (38), including uterine position, fibroid position, fibroid volume, fibroid type (subserous, intramural, and submucosal), subcutaneous fat thickness (anterior abdominal wall at the largest level of fibroids), ventral skin distance (the closest distance from the fibroid ventral side to the skin), T2WI signal intensity of fibroids (hypointensity: signal intensity equal to that of the skeletal muscle; isointensity: signal intensity higher than that of the skeletal muscle but lower than that of the myometrium; hyperintensity: signal intensity similar to or higher than that of the myometrium), T2WI signal homogeneity of fibroids, and contrast enhanced degree of fibroids (mild enhanced: fibroids lower than those of the myometrium; moderate enhanced: fibroids similar to those of the myometrium; obvious enhanced: fibroids higher than those of the myometrium).

### 2.4 Analysis of IVIM-DWI images

The IVIM-DWI images were transferred to the dedicated medical image processing software (China, Unite Imaging Healthcare, uWS-MR, R005). Pixels to be processed were manually selected in the image area with a b value of 0 s/mm^2^ to remove the background. Since a b value of 200 s/mm^2^ has been reported (39) to be the appropriate b value for separating the attenuation and diffusion attenuation of microcirculation, it has been designated as the threshold b value. Then, the computer automatically generates IVIM-DWI parameters and the corresponding parametric maps. The IVIM-DWI parameters include D (diffusion coefficient), D^*^ (pseudo-diffusion coefficient), and f (the perfusion fraction). Relative blood flow (rBF) was obtained by multiplying f by D^*^.

**Figure 1:**
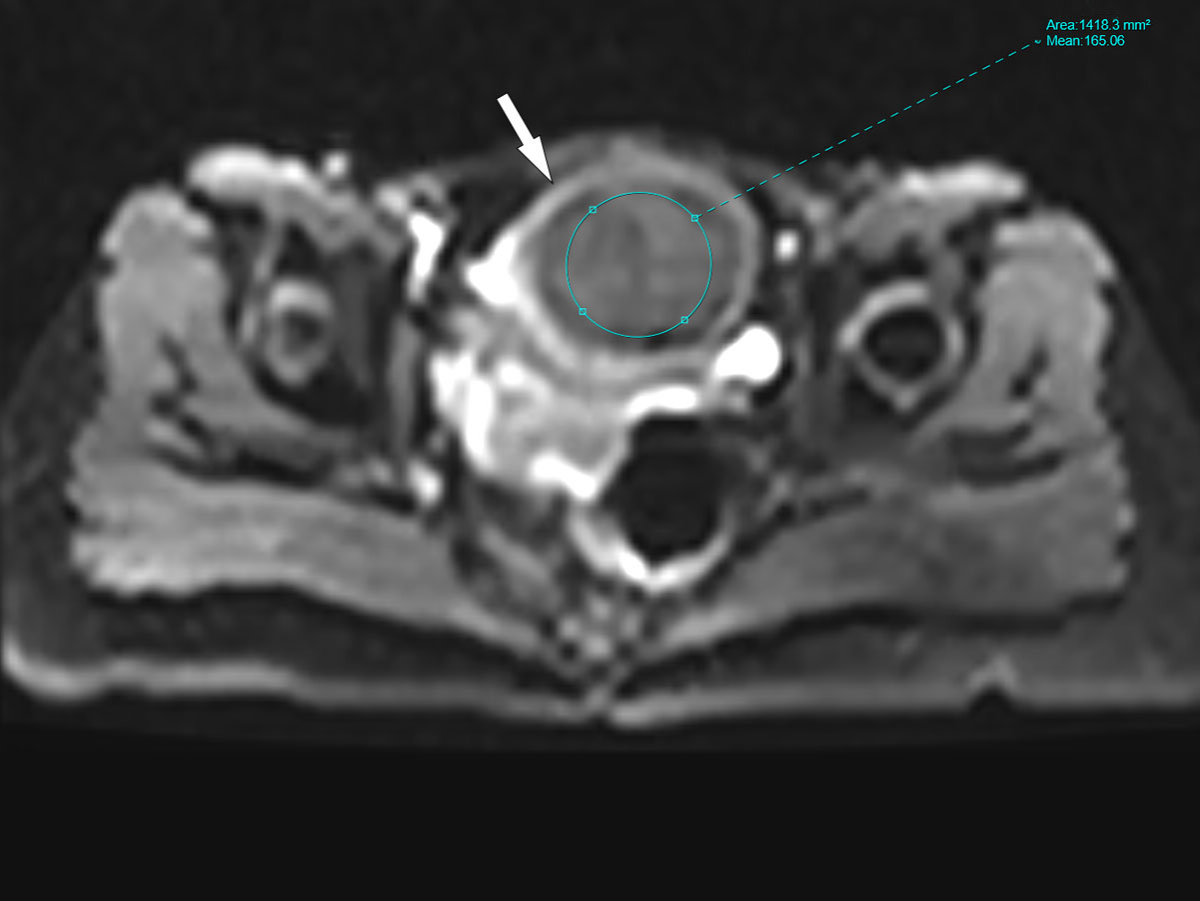
The region of interest (ROI) diagrammatic sketch. The white arrow in the figure refers to uterine fibroid, and the size of ROI covered at least 1/3 of fibroid areas.

Two radiologists with 5 and 3 years of experience in body MR imaging performed the quantitative analyses of IVIM-DWI images to assess inter-reader reproducibility. They were blinded to grouping and clinical data of uterine fibroids, and randomly analyzed the images. Freehand regions of interest (ROIs) were drawn by radiologists’ hands in uterine fibroids on three consecutive sections centered on the largest section of the fibroid. The ROIs were defined by avoiding cystic changes, hemorrhage, and extensive necrosis. The ROI sizes covered at least 1/3 of fibroid areas (**Figure 1**). As a representative value, the average of the three ROIs measurements for each parameter was calculated using the processing software.

### 2.5 Statistical analysis

Reproducibility of individual parameters by radiologists was assessed by calculating the intraclass correlation coefficient (ICC) and ICC ≥ 0.75 indicates good reliability. Based on normality of data distribution, continuous variables were analyzed using the independent sample t-test or the Mann-Whitney U test. Categorical variables were analyzed using the chi square test or the Wilcoxon’s rank sum test. Then, a multivariate logistic regression model was constructed using the statistically significant variables from the different analyses to comprehensively evaluate the predictors of the efficacy of HIFU ablation. The receiver operating characteristic (ROC) curve was drawn, and the area under the ROC curve (AUC), sensitivity, and specificity were calculated to assess the performance of IVIM-DWI parameters in predicting the efficacy of ablation. A nomograph was constructed to visualize the prediction model. The Hosmer-Lemeshow (H-L) test and calibration curves were used to evaluate the fitting of the model. *p*<0.05 was set as the threshold for significance and all analyses were performed using the SPSS (IBM SPSS Statistics 27) and R (R 4.2.2) software.

## 3 Results

### 3.1 Baseline characteristics

The demographic and baseline characteristics of the sufficient ablation and insufficient ablation groups are shown in **Table 1**. The effects of fibroid positions (χ^2 = ^6.88, *p*=0.009), ventral skin distance (χ^2 = ^5.92, *p*=0.015), T2WI signal intensity of fibroids (χ^2 = ^13.66, *p*=0.001), and contrast enhanced degree of fibroids (χ^2 = ^12.31, *p*=0.002) on HIFU efficacy were significant. The ablation effects of uterine fibroids with fibroid position located in anterior wall of the uterus, close to the ventral skin, low hypointensity on T2WI, and mildly enhanced when contrast enhanced were better. However, other factors have little influence on the efficacy of HIFU (*p*>0.05).

**Table 1:** Demographic and baseline data of participants.

Characteristics	Sufficient ablation group	Insufficient ablation group	Value	*p*
N	51	38		
Age (y)	42.7 ± 0.9	44.8 ± 0.9	Z=1.76	0.078
Uterine position (%)			χ^2 = ^0.40	0.527
Anterior position	33(64.7)	27(71.1)		
Posterior position	18(35.3)	11(28.9)		
Fibroid position (%)			χ^2 = ^6.88	0.009^*^
uterus anterior wall	38(74.5)	18(47.4)		
uterus posterior wall	13(25.5)	20(52.6)		
Fibroid volume(cm^3^)	36.9(16.9-79.5)	53.8(30.8-85.1)	Z=1.81	0.071
Fibroid type (%)			χ^2 = ^0.77	0.681
Subserous	2(3.9)	2(5.3)		
Intramural	47(92.2)	33(86.8)		
Submucosal	2(3.9)	3(7.9)		
Subcutaneous fat thickness (%)			χ^2 = ^1.67	0.197
<24.85mm	28(54.9)	26(68.4)		
≥24.85mm	23(45.1)	12(31.6)		
Ventral skin distance (%)			χ^2 = ^5.92	0.015^*^
<45.41mm	25(49.0)	9(23.7)		
≥45.41mm	26(51.0)	29(76.3)		
T2WI signal intensity (%)			χ^2 = ^13.66	0.001^*^
Hypointensity	28(54.9)	7(18.4)		
Isointensity	15(29.4)	15(39.5)		
Hyperintensity	8(15.7)	16(42.1)		
T2WI signal homogeneity (%)			χ^2 = ^1.41	0.234
Homogeneous	36(70.6)	31(81.6)		
Inhomogeneous	15(29.4)	7(18.4)		
Contrast enhancement degree (%)			χ^2 = ^12.31	0.002^*^
Mild enhancement	20(39.2)	4(10.5)		
Moderate enhancement	23(45.1)	18(47.4)		
Obvious enhancement	8(15.7)	16(42.1)		

T2WI, T2-weighted imaging; ^*^ *p*<0.05.

### 3.2 Intraclass correlation coefficients

The ICC were 0.951 (95% confidence interval (CI): 0.927, 0.968) for D, 0.917 (95% CI: 0.876, 0.944) for D^*^, 0.854 (95% CI: 0.778, 0.904) for f, and 0.808 (95% CI: 0.707, 0.874) for rBF, implying good reliability between observers (*p*<0.001) (**Table 2**). Therefore, results of the first observer were used in this study.

**Table 2:** Intraclass correlation coefficients between two observers on IVIM-DWI parameters D, D^*^, f, and rBF.

Parameters	ICC	95% CI	*p*
D	0.951	0.927, 0.968	<0.001
D^*^	0.917	0.876, 0.944	<0.001
f	0.854	0.778, 0.904	<0.001
rBF	0.808	0.707, 0.874	<0.001

IVIM-DWI, intravoxel incoherent motion diffusion-weighted imaging; ICC, intraclass correlation coefficient; CI, confidence interval; D, diffusion coefficient; D^*^, pseudo-diffusion coefficient; f, the perfusion fraction; rBF, relative blood flow.

### 3.3 The IVIM-DWI parameters of two groups

**Figure 2:**
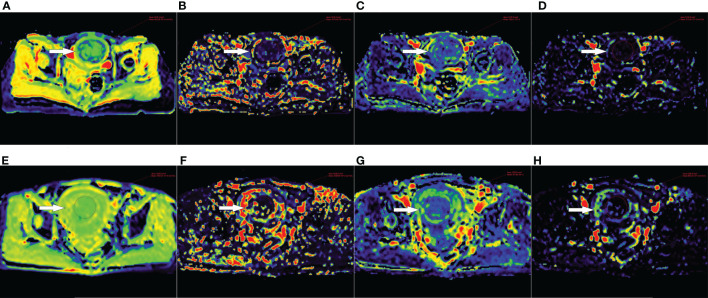
**(A–D)** D, D*, f and rBF maps of a 47-year-old patient with uterine fibroid (white arrow) in the sufficient ablation group. **(E–H)** D, D*, f and rBF maps of a 46-year-old patient with uterine fibroid (white arrow) in the insufficient ablation group.

The IVIM-DWI parameters and the corresponding parametric maps of the sufficient ablation and insufficient ablation groups are shown in **Table 3** and **Figures 2A–H**. The D value of the sufficient ablation group (931.0(851.5-987.4) × 10^−6^ mm^2^/s) was significantly lower than that of the insufficient ablation group (1052.7(1019.6-1158.7) × 10^−6^ mm^2^/s) (*p*<0.001). However, differences in D^*^, f, and rBF values between the groups were all insignificant (*p*>0.05).

**Table 3:** The IVIM-DWI parameters of the sufficient ablation and insufficient ablation groups.

Parameters	Sufficient ablation group	Insufficient ablation group	Z	*p*
D (10^−6^ mm^2^/s)	931.0(851.5-987.4)	1052.7(1019.6-1158.7)	5.143	<0.001
D^*^ (10^−5^ mm^2^/s)	3945.6(2934.7-6032.3)	3666.1(2778.6-4509.7)	1.526	0.127
f (10^−3^%)	160.5(127.8-179.0)	153.4(136.3-185.6)	0.008	0.993
rBF (10^−5^ mm^2^/s)	588.5(448.4-1017.5)	622.9(379.0-804.5)	0.912	0.362

IVIM-DWI, intravoxel incoherent motion diffusion-weighted imaging;

D, diffusion coefficient;

D^*^, pseudo-diffusion coefficient;

f, the perfusion fraction;

rBF, relative blood flow.

### 3.4 Effects of IVIM-DWI parameters and imaging factors on HIFU efficacy prediction

**Figure 3:**
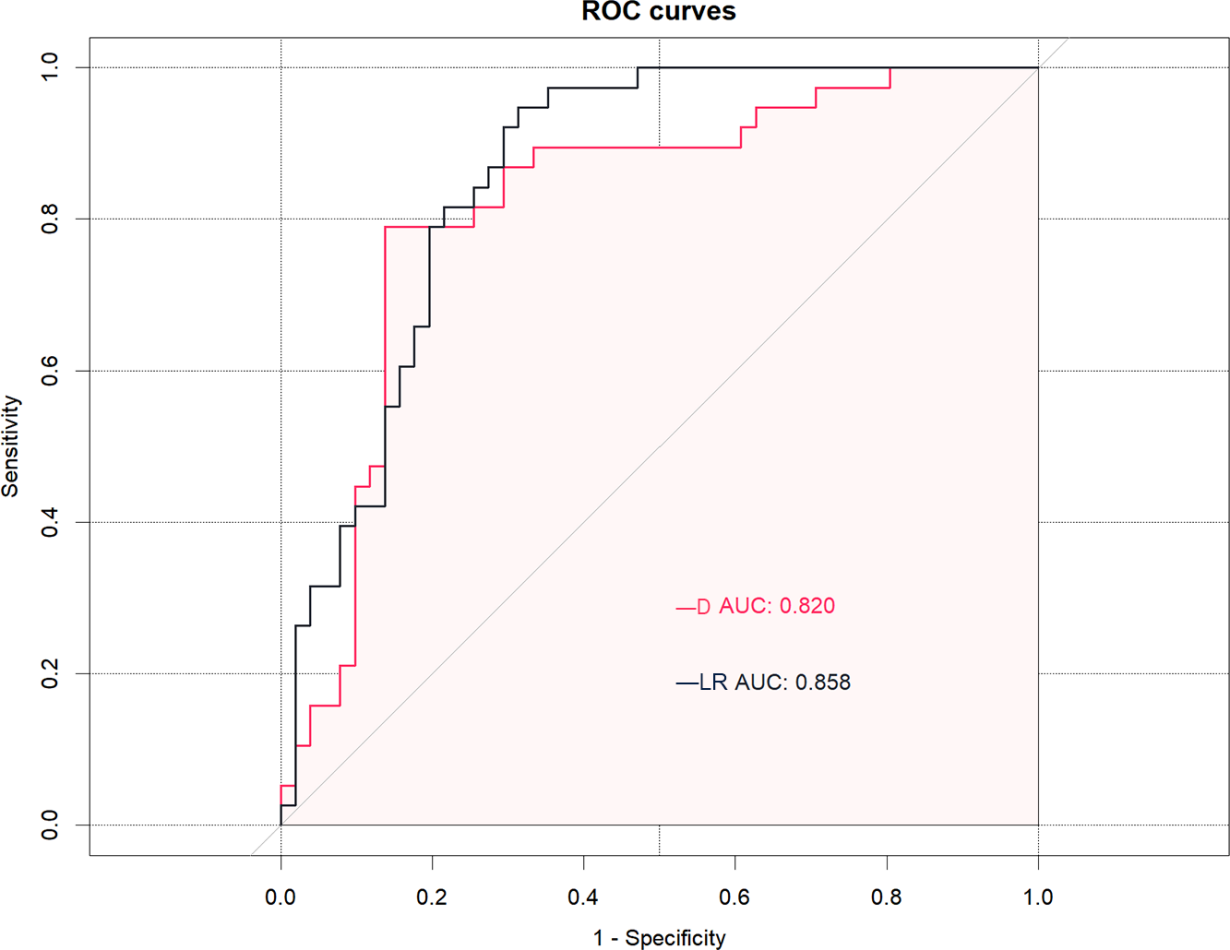
The receiver operating characteristic (ROC) curves analysis of the D (diffusion coefficient) value and logistic regression (LR) model for predicting the effects of high-intensity focused ultrasound (HIFU). The D value and LR model demonstrated equally good prediction performance, with an AUC of 0.820 in the D value and an AUC of 0.858 in the LR model.

**Figure 4:**
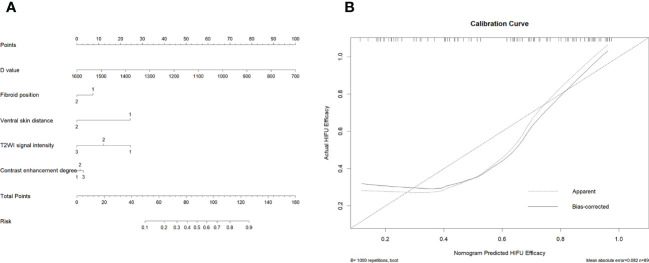
Establishment and performance of the logistic regression model. **(A)** The logistic regression model was utilized to develop a nomogram. **(B)** Calibration curves of the nomogram. The x-axis represents the high-intensity focused ultrasound (HIFU) efficacy predicted by logistic regression model, and the y-axis represents the actual HIFU efficacy.

**Table 4** and **Figure 3** show that the cut-off value, AUC value, specificity and sensitivity of the IVIM-DWI parameter D value were 1008.2 × 10^-6^ mm^2^/s, 0.820 (95% CI: 0.728, 0.912), 0.863 and 0.789, respectively. The logistic regression model was constructed with the D value, fibroid position, ventral skin distance, T2WI signal intensity of fibroids, and contrast enhanced degree of fibroids. The AUC value, cumulative value, specificity and sensitivity of the logistic regression model were 0.858 (95%CI: 0.781, 0.935), 0.686 and 0.947, respectively. Although the AUC value of the logical regression model was higher than that of only the IVIM-DWI parameter D value, there was no significant difference between them (*p*=0.278). The goodness of fit effect of the logistic regression model is good (*p*=0.612). The nomogram is a convenient visible tool for estimating the risk of variables (**Figure 4A**). The calibration curves revealed a good consistency between the actual and predicted effects (**Figure 4B**).

**Table 4:** Performance of IVIM-DWI quantitative parameters in predicting the efficacy of HIFU ablation of uterine fibroids.

	Cut-off (10^−6^ mm^2^/s)	AUC (95% CI)	Specificity	Sensitivity
D	1008.2	0.820 (0.728, 0.912)	0.863	0.789
Logistic regression model	-	0.858 (0.781, 0.935)	0.686	0.947

IVIM-DWI, intravoxel incoherent motion diffusion-weighted imaging; HIFU, high-intensity focused ultrasound; AUC, area under the receiver operating characteristic curve; CI, confidence interval; D, diffusion coefficient.

## 4 Discussion

The IVIM-DWI can distinguish between perfusion and diffusion, and it can be used for non-invasive quantitative assessment of microcirculation in capillaries and molecular diffusion. The IVIM-DWI quantitative parameters are potential alternatives for contrast enhanced MRI in assessing the efficacy of HIFU ablation of uterine fibroids to a certain extent. We found that high D value indicates poor HIFU efficacy. In addition, the logistic regression model established by the D values, fibroid positions, ventral skin distance, T2WI signal intensity of fibroids, and contrast enhanced degree of fibroids had a good predictive performance. These findings suggest that the IVIM-DWI quantitative parameters can be used as potential imaging markers to predict the efficacy of HIFU ablation of uterine fibroids before surgery. It helps patients and their doctors to make smarter decisions, which ensures the success rate of HIFU treatment and reduced medical costs.

The HIFU principle is that the ultrasound focuses deep within the tumor under the guidance of the monitoring image, quickly increasing the local tissue temperature and inducing coagulative necrosis (40, 41). The outcomes of HIFU ablation of uterine fibroids are affected by many factors. A higher NPVR after uterine fibroid ablation treatment is associated with symptomatic relief and reduced fibroid volume (34). The MRI is an important tool for predicting NPVR before HIFU operation. We found that fibroid positions, ventral skin distance, T2WI signal intensity of fibroids, and contrast enhanced degree of fibroids are the main clinical influencing factors of NPVR. The ablation effects of uterine fibroids with fibroid position located in anterior wall of the uterus, close to the ventral skin, low hypointensity on T2WI, and mildly enhanced when contrast enhanced were better, that is, higher NPVR. This can be explained by the following facts. During ultrasonic transmission, energy attenuation is generated due to refraction, reflection, absorption and scattering (33, 42). Therefore, compared with fibroids on the back wall or near sacrum, energy attenuation of fibroids on the front wall or close to the ventral skin may be less, and its ablation effect better. This is consistent with a previous finding (42). The number of smooth muscle cells in fibroid tissues with low signals on T2WI are less, and collagen fiber amounts are higher, thus, the energy is easier to deposit, and the ablation effect is better (17). This is consistent with previous research results (18, 31, 42). Fibroids with high blood perfusion levels lose energy continuously due to the blood flow carrying away the energy that the fibroids obtain (21). Therefore, blood supply to the obvious enhancement fibroid is abundant, more energy is lost, and the ablation effect is poor (22, 26). This is consistent with previous research results (31, 42). However, our results slightly differ from those of FAN et al. (38). They reported that uterine fibroids of hypointense on T2WI, large sized, mild enhancement on enhanced scanning, fibroids in anterior walls of the uterus, anteverted uterus, and short distance from the ventral side of fibroid to the skin can all be easily ablated, with better ablation effects. This difference may be because they used a linear regression model to analyze the factors that affect efficacy, while we used a logistic regression model for analysis, which may narrow the differences between some influencing factors. Moreover, we include all eligible patients with single fibroid and multiple fibroids, thus obtaining more clinical information, which makes the independent variable information richer. However, in the study of FAN et al., they only investigated patients with single fibroid, which may miss some information about the patients, resulting in selection bias. This may be the reason why the results of the two studies are slightly different.

We analyzed the correlation between IVIM-DWI quantitative parameters and the efficacy of HIFU ablation of uterine fibroids to non-invasively assess the diffusion and perfusion characteristics of uterine fibroids without using contrast agents, rather than just basing our conclusions on conventional MR images. We found that high D value indicates poor HIFU efficacy. That is because fibroids with high D values imply increased extracellular interstitial water levels and/or increased blood volume, and poor ablation effects (31). At present, most studies use the ADC value of DWI to non-invasively assess the diffusion of uterine fibroids. Sainio et al. (43) found that a lower ADC value before HIFU ablation of uterine fibroids is associated with higher NPVR. They also suggested that ADC classification might be superior to the traditional Funaki classification in efficacy prediction. However, it has not been established how the ADC value affects thermal ablation. Verpalen et al. (24) found that the ADC value can distinguish between uterine fibroids and myometrium, and between the different types of uterine fibroids, which may be a useful tool for efficacy prediction. Liao et al. (30) found that the average ADC value decreased after HIFU ablation treatment. They also observed the phenomenon of a high signal loop on DWI images, and found that if the high signal loop was complete, it could replace enhanced scanning in assessing fibroid volume and HIFU ablation effects. Compared with the ADC value, the D value obtained from high b value can accurately reflect the cell density, which should be investigated further. In addition, the D^*^, f, and rBF values were not significantly different in this study, which may be attributed to the relatively high sensitivity of D^*^ and f values of body DWI to noise (44). Moreover, this study explored early efficacy of HIFU ablation in the treatment of uterine fibroids (immediate NPVR). We performed MRI scans and patient grouping shortly after treatment. However, the observation period was short, and the early tissue was mainly swollen, which may not have affected the vascular microcirculation perfusion of the tissue. We speculate that after prolonging the follow-up time, the perfusion parameters (D^*^, f) may be more clinically significant as an index to predict the efficacy of HIFU. To further investigate the complementary value of IVIM-DWI quantitative parameters in predicting the efficacies of HIFU ablation for uterine fibroids, we established a logistic regression model by combining conventional MR parameters and IVIM-DWI quantitative parameters.

This study has certain limitations. First, this was a retrospective study that only included uterine fibroid patients who chose HIFU ablation therapy by themselves, which may result in selection bias. Second, our sample size was small, which limits the statistical power of our findings. Large sample studies should be performed to verify our results. Third, our study may have been subject to biases as we could not compare our findings with histological data. This is because the diagnosis of uterine fibroids is not based on histological analysis, but on clinical and imaging results. However, this limitation is insurmountable considering the clinical practice.

## 5 Conclusions

The IVIM-DWI quantitative parameters can be used as potential imaging markers for non-invasive prediction of the early effects of preoperative HIFU ablation for uterine fibroids. A high D value before treatment may indicate that the treatment will be less effective in the early stages.

## Data availability statement

The original contributions presented in the study are included in the article/supplementary materials, further inquiries can be directed to the corresponding author/s.

## Ethics statement

The studies involving human participants were reviewed and approved by the Ethics Committee of Affiliated Hospital of North Sichuan Medical College. Written informed consent for participation was not required for this study in accordance with the national legislation and the institutional requirements.

## Author contributions

YJ, SQ and XH contributed to conception and design of the study. YW and YL organized the database. YJ and SQ performed the statistical analysis. YJ, SQ and XH wrote the first draft of the manuscript. NL, LT, JF and QJ wrote sections of the manuscript. All authors contributed to the article and approved the submitted version.
